# Effect of SiC Concentration on the Microstructure and Anti-Wear Performance of Electrodeposited Ni-SiC Composite Coatings Constructed for Piston Ring Application

**DOI:** 10.3390/ma18051117

**Published:** 2025-03-01

**Authors:** Fengwu Zhang, Qiuhua Wang, Huajie Shen, Caixia Bai, Chaoyu Li, Dehao Tian, Baojin Wang

**Affiliations:** 1 School of Mechanical and Electrical Engineering, Sanming University, Sanming 365004, China; 20230368@fjsmu.edu.cn; 2 Yunnan Key Laboratory of Forest Disaster Warning and Control, College of Civil Engineering, Southwest Forestry University, Kunming 650224, China; qhwang2010@swfu.edu.cn; 3 School of Design, Fujian University of Technology, Fuzhou 350118, China; shenhuajie@fjut.edu.cn (H.S.); 2242007004@fjut.edu.cn (C.B.); 4 School of Mechanical Science and Engineering, Northeast Petroleum University, Daqing 163318, China; bjwangbaojin@nepu.edu.cn

**Keywords:** Ni-SiC composite coatings, electrodeposition, microstructure, anti-wear performance, piston ring

## Abstract

At present, the improvement of anti-wear performance of piston rings remains a challenge. In this article, Ni-SiC composite coatings fabricated at 3, 9, and 15 g/L SiC were denoted as NSc-3, NSc-9, and NSc-15 coatings. Meanwhile, the influence of SiC concentration on the surface morphology, phase structure, microhardness, and anti-wear performance of electrodeposited Ni-SiC composite coatings were investigated utilizing scanning electron microscopy, X-ray diffraction, a microhardness tester, and a friction–wear tester, respectively. The SEM images presented NSc-9 coatings with a compact, flat, or cauliflower-like surface morphology. The cross-sectional morphology and EDS results showed that the Si and Ni elements were uniformly distributed in the NSc-9 coatings with dense and flat microstructures. Moreover, the average grain size of the NSc-9 coatings was only 429 nm. Furthermore, the microhardness and indentation path of the NSc-9 coatings were 672 Hv and 13.7 μm, respectively. Also, the average friction coefficient and worn weight loss of the NSc-9 coatings were 0.46 and 29.5 mg, respectively, which were lower than those of the NSc-3 and NSc-15 coatings. In addition, a few shallow scratches emerged on the worn surfaces of the NSc-9 coatings, demonstrating their outstanding anti-wear performance when compared to the NSc-3 and NSc-15 coatings.

## 1. Introduction

At present, the automotive industry is becoming productive and prosperous in the foreign and domestic markets, producing a burst of innovative vehicles. However, the automotive industry is still plagued by some issues, such as the anti-wear performance and service life of combustion engines [1,2,3]. The composition of combustion engines includes engine cylinders, plungers, piston rings, bearings, valves, and so on. Among these components, the friction system between engine cylinders and piston rings has a significant influence on the properties and efficiency of the engine [4]. Generally, chromium is deposited on the surface of piston rings using the electrodeposition method to enhance the anti-wear performance and service life [5]. However, during the plating chromium process, the electrolyte containing Cr^6+^ ions can cause environmental pollution and personal injury [6]. Therefore, to reduce the damage to the environment and to people, the intensive search for alternative materials to chromium employed for the protection of piston rings in the world is emerging.

Nickel-based coatings are considered ideal materials to replace chromium due to their advantages of outstanding mechanical performance, excellent abrasion, and corrosion resistance [7]. For example, Kucharska et al. [8] found that Ni-Al_2_O_3_ composite coatings via ultrasonic electrodeposition had the largest corrosion potential of −199 mV. Ali et al. [9] found that Ni-AlN composite coatings fabricated at 9 g/L AlN had the lowest corrosion current of 1.29 μA/cm^2^. Yang et al. [10] proposed that Ni-TiN composite coatings produced at a 7 mm nozzle diameter possessed a microhardness of 647 Hv. Ji et al. [11] reported that Ni-SiC composite coatings manufactured at 1 g/L SiC and by using rotating magnetic field-assisted electrodeposition had the highest microhardness of 653 Hv. Among these coatings, SiC, which features high microhardness and excellent stability, could be embedded into a nickel matrix to provide protection for steel substrates; this has attracted the attention of domestic and foreign scholars [12]. For instance, Islam et al. [13] prepared Ni and Ni-P-SiC composite coatings via the electroless method. They concluded a two-fold increase in the microhardness of Ni-P-SiC composite coatings compared to pure Ni coatings. Ying et al. [14] used the electroless approach for producing Ni-P-SiC and Ni-P-SiC-WS_2_ composite coatings. They found that the addition of WS_2_ could obviously enhance the tribological properties and anti-corrosion ability of Ni-P-SiC composite coatings. Farzaneh et al. [15] prepared Ni-P-SiC nanocoatings via the electroless method. They investigated the effect of SiC size on the microstructure and performance of Ni-P-SiC composite coatings. However, the electroless Ni-P-SiC composite coatings had faults of slow deposition rate, difficult control, and poor solution stability, leading to their restricted industrial application. Hence, some researchers began experimenting with an electrodeposition technique used for preparing Ni-SiC composite coatings. For example, Gyawali et al. [16] studied the additive of cetyltrimethyl ammonium bromide and saccharin on the microstructure and corrosion resistance of electrodeposited Ni-SiC composite coatings. They found that saccharin could refine grain size and microhardness and reduce the anti-corrosion ability of Ni-SiC composite coatings. Cui et al. [17] discussed the influence of current density on the corrosion resistance of Ni-SiC composite coatings applied in the storage tank field. They concluded that the anti-corrosion performance of Ni-SiC composite coatings manufactured at 2.5 A/dm^2^ was excellent. Yan et al. [18] produced Ni-MoS_2_, Ni-SiC, and Ni-MoS_2_-SiC composite coatings through the electrodeposition approach. They reported that the Ni-MoS_2_ composite coatings had a low friction coefficient, while the Ni-SiC composite coatings had a low wear rate. This research demonstrated that electrodeposited Ni-SiC composite coatings have excellent anti-wear/corrosion performance and that attempts could be made to apply them in the piston ring field. However, research on the effect of SiC concentration on the anti-wear performance of electrodeposited Ni-SiC composite coatings applied in the piston ring field was not found.

Therefore, in this paper, the surface morphology, phase structure, microhardness, and anti-wear performance of Ni-SiC composite coatings fabricated at different SiC concentrations were analyzed in detail. In addition, the worn mechanism of electrodeposited Ni-SiC composite coatings was also explored as well as their application in piston rings.

## 2. Materials and Methods

### 2.1. Preparation

**Figure 1:**
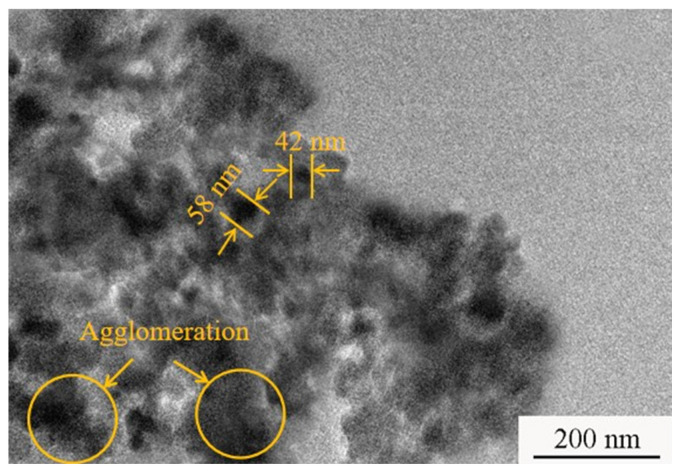
TEM image of SiC nanoparticles.

**Figure 2:**
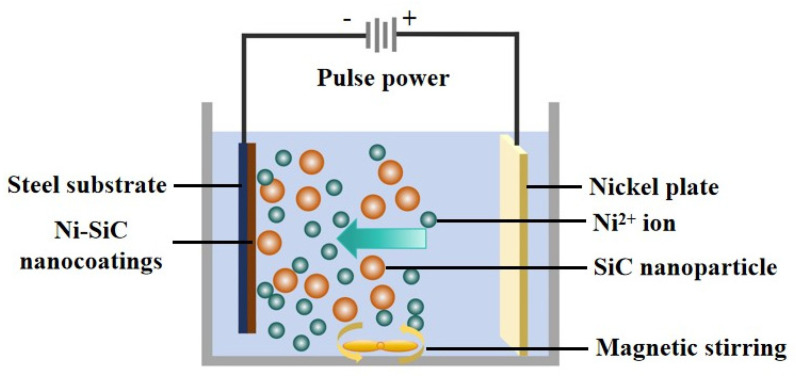
Schematic graph of electrodeposited Ni-SiC composite coatings.

The 1045 steel is a common material used in the piston ring industry. Therefore, in this paper, a 1045 steel substrate with a size of 30 × 50 × 3 mm^3^ and a nickel plate (purity > 99.8%) with dimensions of 40 × 70 × 3 mm^3^ were employed as a cathode and an anode, respectively. SiC nanoparticles with an average size of 40 nm were purchased from Daqing Yutai Science & Technology Co., Ltd. (Daqing, China). A TEM image of SiC nanoparticles is shown in Figure 1. Before pulse electrodeposition, the substrates were firstly treated with abrasive papers with different grits (200, 600, and 1000 grit grade) to achieve a roughness of 0.1 μm. Afterward, the treated substrates were disposed of with a rust agent and an oil remover. Finally, the anode and cathode were placed in the plating solution to prepare the Ni-SiC nanocoatings. A schematic graph of electrodeposited Ni-SiC nanocoatings is presented in Figure 2. The composition of the plating solution is displayed in Table 1. During the electrodeposition process, the plating solution should be disposed of with magnetic stirring to inhibit the agglomeration and deposition of the SiC nanoparticles. The Ni-SiC composite coatings fabricated at 3, 9, and 15 g/L SiC are denoted as NSc-3, NSc-9, and NSc-15 coatings. The operation parameters for fabricating electrodeposited Ni-SiC composite coatings are listed in Table 2. During the electrodeposition process, the CTAB could reduce the interfacial tension of the electrolyte and the porosity, resulting in the improved microstructure and properties of the coatings. The Gugliemi theory illustrates that the electrodeposition process could be dealt with in the following two steps [19]: (1) the electrical field force pushes the SiC nanoparticles with nickel ions to the cathode surface; (2) the SiC nanoparticles and nickel ions co-deposit on the cathode surface and form the Ni-SiC composite coatings.

### 2.2. Characterization

The surface morphology, worn surface morphology, and element content of the Ni-SiC composite coatings were determined using an S3400-type scanning electron microscope (HITACHI, Tokyo, Japan) and an INCA-style energy dispersive X-ray (EDX) detector (Oxford Instruments, Oxford, UK), respectively. Furthermore, the phase structure of the Ni-SiC composite coatings was examined utilizing a D-Max/2500-type X-ray diffraction instrument (Rigaku Corporation, Tokyo, Japan). The X-ray source and scanning rate were selected as Cu Kα radiation and 0.02°/s, respectively. According to Scherrer’s formula, the grain size (*D*) of the Ni-SiC composite coatings was calculated as follows:

(1) $$D=\frac{0.89\lambda }{\beta cos\theta }$$

where *λ* represents the X-ray’s wavelength, *β* is the half-width of the diffraction peaks, and *θ* expresses the Bragg’s angle, respectively.

In addition, the indentation depth and microhardness of the Ni-SiC nanocoatings were measured employing a TI-950-style in situ tester with an applied load of 500 μN, a diamond indenter, and a duration time of 15 s, respectively. According to a previous study [20], the microhardness of the Ni-SiC composite coatings was recorded at five random positions and averaged as the final microhardness. The standard deviation of the measured Ni-SiC composite coatings was in the range of ±20 Hv. The microhardness (*Hv*) of the Ni-SiC composite coatings was determined using Equation (2) as follows:

(2) $$Hv=(1.8544\times F)/d^{2}$$

where *F* represents the vertical load, and *d* is the diagonal length of the indentation.

**Figure 3:**
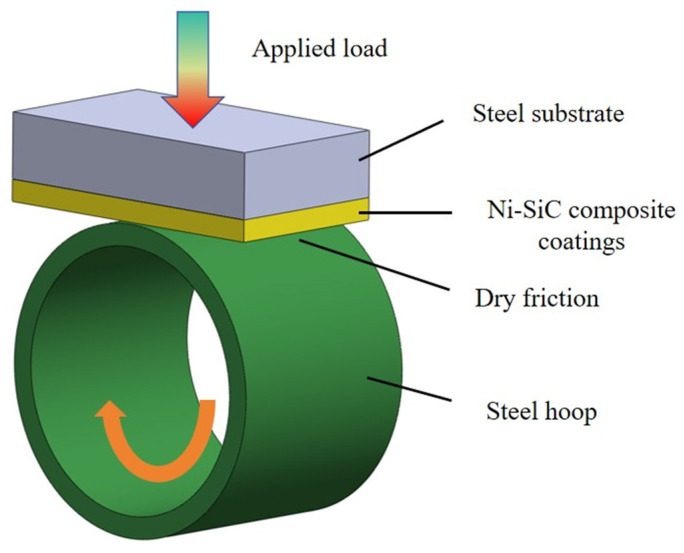
Schematic facility for measuring the anti-wear performance of Ni-SiC composite coatings.

The MRH-6-style friction and wear tester was used to evaluate the anti-wear performance of the Ni-SiC composite coatings. The testing conditions were recorded as follows: an applied load of 5 N, a hard steel hoop (GC15), a wear speed of 0.1 m/s, an indoor temperature, dry sliding conditions, and a wear time of 30 min, respectively. Figure 3 displays the schematic facility for testing the anti-wear performance of the Ni-SiC composite coatings. After a friction and wear test, the worn weight loss (*M*_0_) of the Ni-SiC composite coating could be calculated using Equation (3):

(3) $$M_{0}=M_{1}-M_{2}$$

where *M*_1_ and *M*_2_ are the weight of the Ni-SiC nanocoatings before and after the friction–wear test, respectively.

## 3. Results and Discussion

### 3.1. Surface Morphology Observation

**Figure 4:**
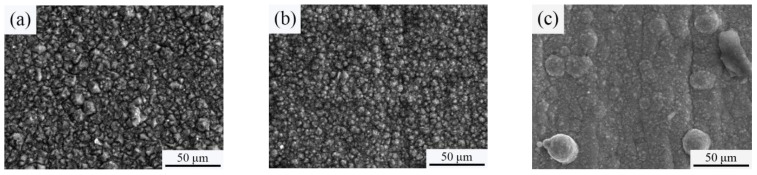
SEM images of Ni-SiC composite coatings manufactured at various SiC concentrations: (**a**) NSc-3, (**b**) NSc-9, and (**c**) NSc-15 coatings.

Figure 4 reveals the surface morphology of a Ni-SiC composite coating prepared at different SiC concentrations. The specific EDX results of the Ni-SiC composite coatings are displayed in Table 3. The observed SEM image of the NSc-3 coatings had a pyramid-like morphology, while the NSc-9 and NSc-15 coatings had a cauliflower-like morphology. Meanwhile, the serious agglomeration of the SiC nanoparticles emerged on the surface of the NSc-15 coatings. Moreover, the Si content of the Ni-SiC composite coatings increased with the growth of the SiC concentration in the plating solution. The variations in the surface morphology and SiC content in the Ni-SiC composite coatings were obviously influenced by the co-deposition rate of the SiC nanoparticles with nickel grains. The electrolyte with a low SiC concentration could not provide sufficient nucleation points and inhibit the grain growth, leading to the NSc-3 coatings having a coarse and uneven surface morphology with a low Si content. The electrolyte with a high SiC concentration increased the viscosity, resulting in the obvious agglomeration of SiC nanoparticles emerging on the surface of the NSc-15 coatings [21]. By contrast, the appropriate SiC concentration could provide sufficient nucleation points and contribute to the co-deposition of SiC nanoparticles with Ni^2+^ ions, forming a dense and smooth surface with a uniform distribution of SiC nanoparticles. Hence, the surface morphology and Si content of the NSc-9 coatings were better than those of the NSc-3 and NSc-15 coatings.

**Figure 5:**
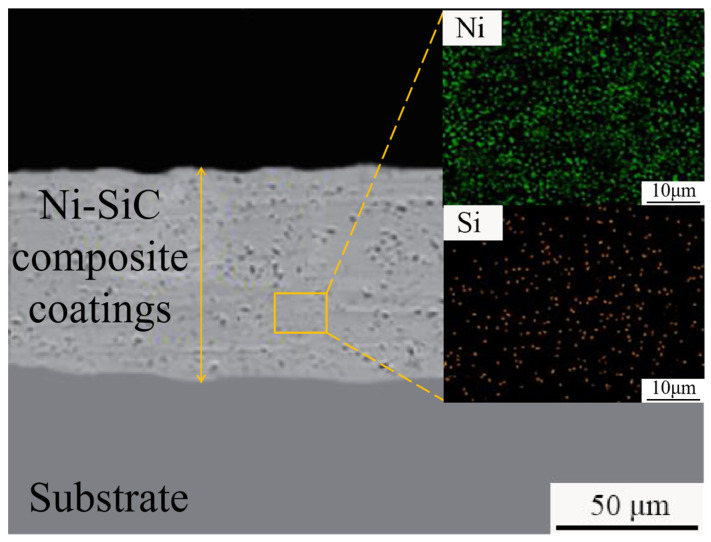
Cross-sectional SEM image and element distribution of NSc-9 coatings.

Figure 5 presents the cross-sectional SEM image and element distribution of a Ni-SiC composite coating prepared at 9 g/L SiC. The cross-sectional surface of the NSc-9 coatings was dense and smooth. Meanwhile, the obvious agglomeration of the SiC nanoparticles was not found in the cross-sectional surface of the NSc-9 coatings. The reason for this phenomenon could be attributed to the suitable SiC concentration favoring nucleation and mass transfer during the electrodeposition process, leading to the internal structure of the Ni-SiC composite coatings becoming compact and flat with a uniform distribution of SiC nanoparticles [22].

### 3.2. XRD Pattern Analysis

**Figure 6:**
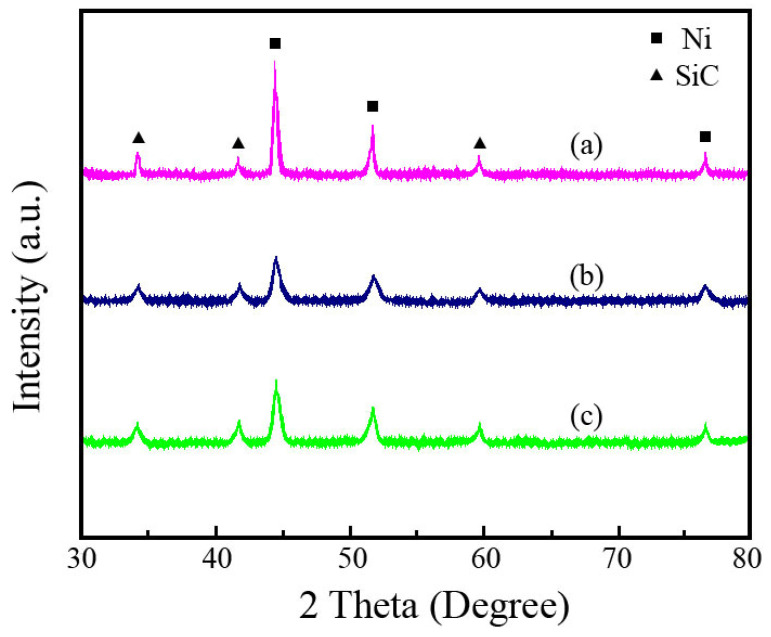
XRD spectra of Ni-SiC composite coatings prepared at different SiC concentrations: (a) NSc-3, (b) NSc-9, and (c) NSc-15 coatings.

The influence of SiC concentration on the XRD patterns of the Ni-SiC composite coatings is presented in Figure 6. The XRD spectra illustrate that the SiC nanoparticles are successfully embedded into the nickel matrix and form Ni-SiC composite coatings. The diffraction peaks of the SiC phase appear at 34.2°, 41.3°, and 59.7°, corresponding to the crystal planes of (111), (200), and (220), respectively [23]. In addition, the diffraction peaks of the nickel grains emerge at 43.6°, 52.9°, and 75.4°, corresponding to the crystal planes of (111), (200), and (220), respectively [24]. After the SiC concentration increased from 3 g/L to 15 g/L, the size of the nickel grains decreased initially and then increased. The sizes of the nickel grains in the NSc-3, NSc-9, and NSc-15 coatings were 874 nm, 429 nm, and 635 nm, respectively. Furthermore, the diffraction peak intensities of the Ni-SiC composite coatings first became wider and then narrowed with the increase in the SiC concentration. This conclusion demonstrated that the most suitable SiC concentration (9 g/L) contributed to the uniform distribution of SiC nanoparticles and refined grain size, which was consistent with the findings proposed by Zheng et al. [25].

### 3.3. Microhardness Value Measurement

**Figure 7:**
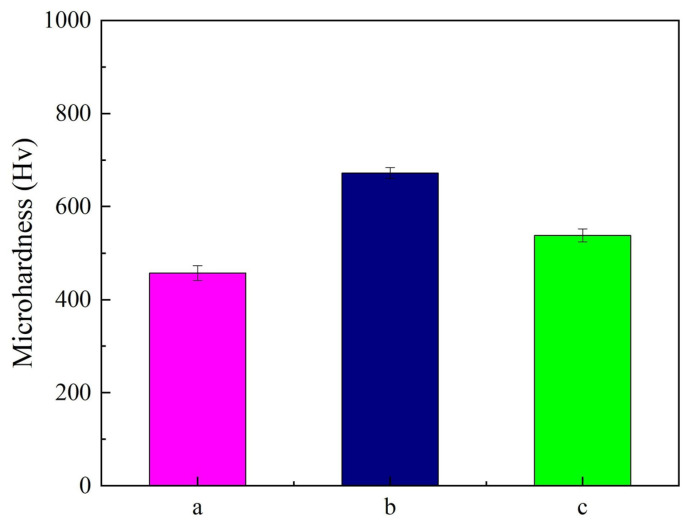
Microhardness value of Ni-SiC composite coatings prepared at different SiC concentrations: (a) NSc-3, (b) NSc-9, and (c) NSc-15 coatings.

Figure 7 illustrates the microhardness of Ni-SiC composite coatings manufactured at various SiC concentrations. It can be seen from the figure that the microhardness of the Ni-SiC composite coatings increased initially and then decreased with the augmentation of the SiC concentration. The microhardness of the NSc-3 coatings, calculated utilizing Equation (2), was only 457 Hv. After the SiC concentration increased to 9 g/L, the microhardness of the NSc-9 coatings increased to 672 Hv. However, the microhardness of the NSc-15 coatings reduced to 538 Hv. The reason behind the microhardness variation could be attributed to the fact that the SiC concentration had an obvious influence on the SiC content, distribution, and grain size [26]. Jiang et al. [27] found that the microhardness of the SiC nanoparticles was higher than that of the nickel matrix, leading to the Ni-SiC composite coatings with a high SiC content to have large microhardness values. However, the agglomerated SiC nanoparticles in the NSc-15 coatings could not form strong dispersion strengthening and fine-grain strengthening, leading to a decrease in the coatings’ compactness and a reduction in the microhardness. By comparison, the uniform distribution of the SiC nanoparticles emerged in the NSc-9 coatings, which generated strong dispersion strengthening and fine-grain strengthening [28]. Hence, the microhardness of the NSc-9 coatings was larger than that of the NSc-3 and NSc-15 coatings.

### 3.4. Anti-Wear Performance Investigation

**Figure 8:**
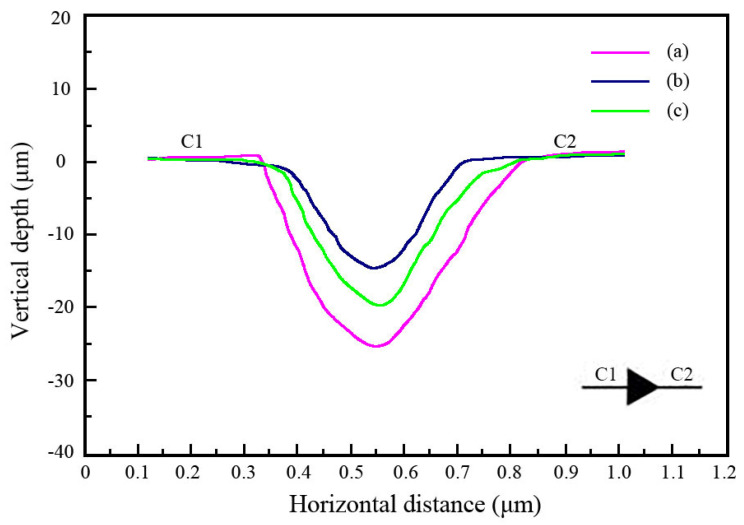
Indentation depths of Ni-SiC composite coatings prepared at different SiC concentrations: (a) NSc-3, (b) NSc-9, and (c) NSc-15 coatings.

Figure 8 demonstrates the indentation depth of the Ni-SiC composite coatings produced at different SiC concentrations. The indentation depths of the NSc-3, NSc-9, and NSc-15 coatings were 24.8 μm, 13.7 μm, and 19.6 μm, respectively. The measured result of the indentation depth could be illustrated by the increasing microhardness and uniform distribution of the SiC nanoparticles [29]. The increasing microhardness could directly decrease the damage from the friction pair. Additionally, the uniform distribution of the SiC nanoparticles could hinder grain boundary migration and reduce the indentation depth. These findings were similar to the report of Ma et al. [30] on the effect of particle size on the indentation depth of Ni-TiC composite coatings.

**Figure 9:**
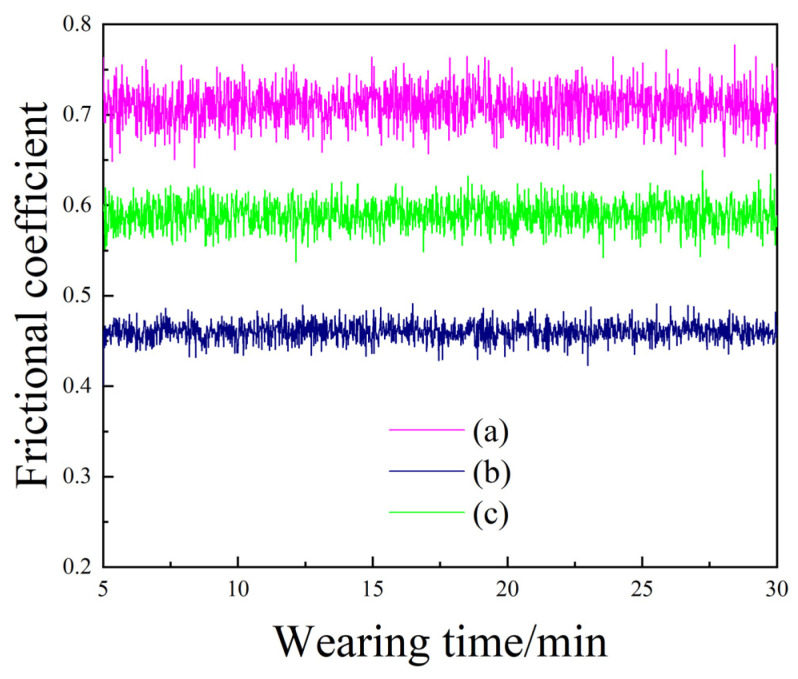
Friction coefficients of Ni-SiC composite coatings prepared at different SiC concentrations: (a) NSc-3, (b) NSc-9, and (c) NSc-15 coatings.

Figure 9 depicts the friction coefficients of Ni-SiC composite coatings fabricated at various SiC concentrations. It can be seen that the friction coefficient of the Ni-SiC composite coatings is highly related to the distribution and size of the SiC nanoparticles on the coatings’ surface. Liu et al. [31] reported that the small friction coefficient could decrease the damage to the alloy ball and enhance the anti-wear performance of the Ni-SiC composite coatings. The large size of the nickel grains and the low SiC content in the NSc-3 and NSc-15 coatings resulted in the average friction coefficients of the composite coatings to be 0.71 and 0.59, respectively. By contrast, the small size of the nickel grains and the high SiC content in the NSc-9 coatings led to the average friction coefficient of the Ni-SiC composite coatings to be reduced to only 0.46. Additionally, the uniform distribution of the SiC nanoparticles in the Ni-SiC composite coatings could significantly decrease the contact area between the coatings and the alloy ball, leading to a reduction in the friction coefficient. In addition, the SiC particles separated from the composite coatings changed the friction mode from sliding friction to rolling friction, which resulted in the prominent reduction in the friction coefficient. This conclusion has been proved by Xia et al. [32] about the variation in the friction coefficient under various friction modes during friction and wear testing. Therefore, the lowest friction coefficient of the Ni-SiC composite coatings, prepared at 9 g/L SiC and illustrating anti-wear performance, was outstanding.

**Figure 10:**
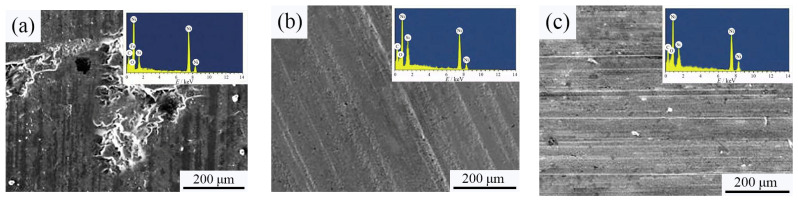
Worn surface morphology of Ni-SiC composite coatings prepared at different SiC concentrations: (**a**) NSc-3, (**b**) NSc-9, and (**c**) NSc-15 coatings.

Figure 10 shows the worn surface morphology of Ni-SiC composite coatings manufactured at various SiC concentrations. The analysis of the observed SEM micrograph presents obvious grooves and pits that appeared on the worn surface of the NSc-3 and NSc-15 coatings, whereas the NSc-9 coatings possessed a few shallow scratches. In addition, the EDX results showed that the Fe element existed on the worn surface of the NSc-3 coatings, which demonstrated that the anti-wear performance of the NSc-3 coatings was worse than that of the NSc-9 and NSc-15 coatings.

**Figure 11:**
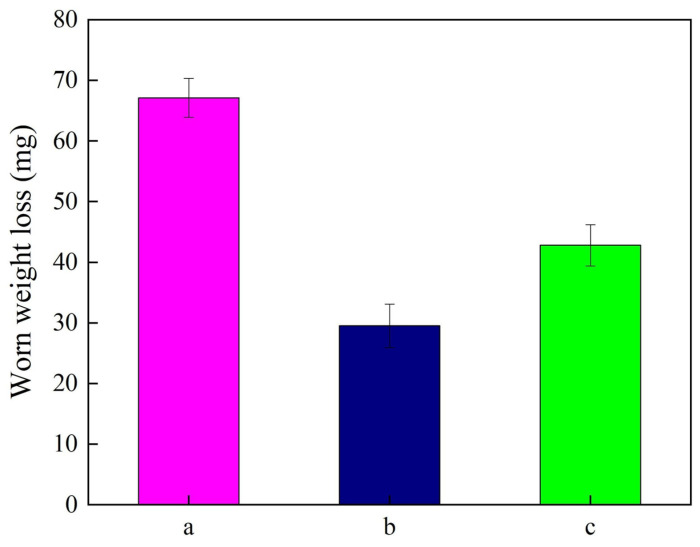
Worn weight losses of Ni-SiC composite coatings prepared at different SiC concentrations: (a) NSc-3, (b) NSc-9, and (c) NSc-15 coatings.

Figure 11 explains the worn weight losses of Ni-SiC composite coatings deposited at various SiC concentrations. The experimental results suggested that the worn weight loss of the Ni-SiC composite coatings decreased initially and then increased with the increase in the SiC concentration. As calculated utilizing Equation (3), the worn weight loss of the NSc-3 coatings was 67.1 mg. After the SiC concentration increased to 9 g/L SiC, the worn weight loss of the Ni-SiC composite coatings reduced to 29.5 mg. However, the worn weight loss of the NSc-15 coatings rose to 42.8 mg.

The observed result could be illustrated considering the effect of SiC concentration on the microstructure and anti-wear performance of the Ni-SiC composite coatings [33]. The NSc-3 coatings with a low SiC content and a large and coarse size of the nickel grains could not effectively resist the damage from the alloy ball. Meanwhile, the NSc-15 coatings had serious agglomeration of the SiC nanoparticles, leading to the SiC nanoparticles being easily separated from the nickel matrix. Furthermore, the large microhardness and low friction coefficient could significantly reduce the worn weight loss of the Ni-SiC composite coatings [34]. In addition, the uniform distribution of the SiC nanoparticles in the NSc-9 coatings generated a strong pinning effect, which could obviously decrease the damage of the alloy ball. A similar wear mechanism of Ni-SiC composite coatings obtained with micro-sized SiC particles has been proposed by Wang et al. [35].

## 4. Conclusions

(1) The SEM images show that a dense, flat microstructure with a fine grain size appeared on the surface of the NSc-9, while the NSc-9 and NSc-15 coatings had a cauliflower-like morphology. Meanwhile, the grain sizes of the NSc-3, NSc-9, and NSc-15 coatings were 874 nm, 429 nm, and 635 nm, respectively.
(2) In comparison to the NSc-3 and NSc-15 coatings, the NSc-9 coatings possessed a maximum microhardness of 672 Hv and a minimum indentation depth of 13.7 μm, demonstrating an excellent anti-wear performance.
(3) Furthermore, the NSc-9 coatings had the lowest friction coefficient of 0.46 and the smallest worn weight loss of 29.5 mg, indicating their outstanding anti-wear performance and their expanded application in piston rings.

## Figures and Tables

**Table 1:** Composition of plating solution for electrodeposition.

Chemical Reagent	Specific
NiSO_4_·6H_2_O (g/L)	240
NiCl_2_·6H_2_O (g/L)	35
CTAB (mg/L)	50
H_3_BO_3_ (g/L)	30
SiC concentration (g/L)	3, 9, and 15

**Table 2:** Operation parameters for obtaining electrodeposited Ni-SiC composite coatings.

Operation Parameters	Specific
Current density (A/dm^2^)	4.5
Electrolyte temperature (°C)	55
Electrodeposition time (min)	40
pH	4.1
Duty cycle (%)	30
Agitation speed (rmp)	600

**Table 3:** EDX results of Ni-SiC composite coatings obtained at different SiC concentrations.

SiC Concentration	3 g/L	9 g/L	15 g/L
Si Content of Composite Coatings	1.9 wt.%	3.2 wt.%	4.5 wt.%

## Data Availability

The original contributions presented in this study are included in the article. Further inquiries can be directed to the corresponding author.
